# Is adrenal adenoma to carcinoma transformation possible?—Illustrative cases and literature review

**DOI:** 10.1210/clinem/dgag022

**Published:** 2026-01-28

**Authors:** Sophie Howarth, James MacFarlane, Lisa Yang, Ines Harper, Ashley Shaw, Yasir S Elhassan, Vasilis Kosmoliaptsis, Mark Gurnell, Cristina L Ronchi, Ruth T Casey

**Affiliations:** Cambridge Endocrine Molecular Imaging Group, Metabolic Research Laboratories, Institute of Metabolic Science, Addenbrooke's Hospital, Hills Road, Cambridge CB2 0QQ, UK; Department of Endocrinology, Cambridge University Hospitals NHS Foundation Trust, Cambridge CB2 0QQ, UK; Cambridge Endocrine Molecular Imaging Group, Metabolic Research Laboratories, Institute of Metabolic Science, Addenbrooke's Hospital, Hills Road, Cambridge CB2 0QQ, UK; Department of Endocrinology, Cambridge University Hospitals NHS Foundation Trust, Cambridge CB2 0QQ, UK; Department of Endocrinology, Cambridge University Hospitals NHS Foundation Trust, Cambridge CB2 0QQ, UK; Department of Radiology, Cambridge University Hospitals NHS Foundation Trust, Cambridge CB2 0QQ, UK; Department of Nuclear Medicine, Cambridge University Hospitals NHS Foundation Trust, Cambridge CB2 0QQ, UK; Department of Radiology, Cambridge University Hospitals NHS Foundation Trust, Cambridge CB2 0QQ, UK; Department of Metabolism and Systems Science, College of Medicine and Health, University of Birmingham, Birmingham B15 2WB, UK; Department of Endocrinology, Queen Elizabeth Hospital, University Hospital Birmingham NHS Foundation Trust, Birmingham B15 2WB, UK; Department of Surgery, Cambridge University Hospitals NHS Foundation Trust, Cambridge CB2 0QQ, UK; Cambridge Endocrine Molecular Imaging Group, Metabolic Research Laboratories, Institute of Metabolic Science, Addenbrooke's Hospital, Hills Road, Cambridge CB2 0QQ, UK; Department of Endocrinology, Cambridge University Hospitals NHS Foundation Trust, Cambridge CB2 0QQ, UK; Department of Metabolism and Systems Science, College of Medicine and Health, University of Birmingham, Birmingham B15 2WB, UK; Department of Endocrinology, Queen Elizabeth Hospital, University Hospital Birmingham NHS Foundation Trust, Birmingham B15 2WB, UK; Cambridge Endocrine Molecular Imaging Group, Metabolic Research Laboratories, Institute of Metabolic Science, Addenbrooke's Hospital, Hills Road, Cambridge CB2 0QQ, UK; Department of Endocrinology, Cambridge University Hospitals NHS Foundation Trust, Cambridge CB2 0QQ, UK; Department of Genetics, University of Cambridge, Cambridge CB2 0QQ, UK

**Keywords:** adrenocortical carcinoma, adrenal adenoma, adrenal incidentaloma

## Abstract

**Context:**

A minority of adrenocortical carcinomas (ACC) demonstrate indolent growth but can usually be identified by radiological characteristics such as inhomogeneity or high unenhanced density. However, cases of ACC developing from a lesion with benign radiological features at baseline and histopathological reports of ACC embedded within an adrenal adenoma have emerged. The adenoma to carcinoma transition in the adrenal gland may be a possible, albeit rare, phenomenon.

**Case Descriptions:**

We present 2 cases from 2 tertiary centers in the United Kingdom who were diagnosed with an adrenal adenoma and showed stable radiological characteristics over 14 years and 11 years, respectively, prior to a rapid enlargement of the lesion and diagnosis with ACC. We review the existing literature and describe 4 additional cases who met radiological criteria for an adrenal adenoma and demonstrated prolonged lesion stability (>5 years) on serial imaging prior to a subsequent diagnosis of ACC. We also identify 4 case reports of patients with radiologically indeterminate adrenal nodules at baseline who demonstrated prolonged size stability for up to 10 years prior to an accelerated growth phase and diagnosis with ACC. There was no significant difference in the average time to ACC diagnosis seen in those with benign radiological features at baseline (10.5 years, n = 6) and those with radiologically indeterminate nodules at baseline (8.75 years, n = 4, *P* = .39).

**Conclusion:**

Adrenal lesions with baseline benign radiological characteristics can very rarely transpire to be ACC. Prolonged dimensional stability cannot be considered a reassuring feature in isolation when assessing radiologically indeterminate adrenal lesions.

The adenoma-carcinoma sequence, in which carcinogenesis results from the progressive accumulation of driver mutations, has been demonstrated in several cancer types and is perhaps best exemplified by colorectal cancer, in which villous and tubulovillous adenomas show a particular propensity to progress to overt malignancy (1). Although it is conceivable that a similar stepwise progression could occur in the adrenal cortex, to date there is little evidence to support such a pathway. Indeed, contemporary clinical practice guiding the management of adrenal lesions relies on the principle that adrenocortical adenomas remain benign and do not transform to malignant disease (2, 3).

Incidental adrenal lesions (“incidentalomas”) detected on cross-sectional computed tomography (CT) imaging have a prevalence between 1.05% and 8.7% depending on the age of the population (4). Benign, nonfunctioning adenomatous or hyperplastic lesions account for 40% to 70% of all incidentalomas (5), and autopsy studies show that adrenal adenomas are common in the wider population with a median frequency of 3% (4). In contrast, adrenocortical carcinoma (ACC) is rare, affecting between 0.5 and 2 per million population per year (6), and diagnosed in 0.4% to 4% of all patients with adrenal incidentalomas (5). ACC is an aggressive cancer with a variable prognosis that is substantially poorer in the presence of metastatic disease (7). Complete surgical resection provides the only means of cure and therefore early identification of malignant adrenal lesions is critical (7). However, this need must be balanced against the opportunity costs associated with investigating and surveilling adrenal incidentalomas in resource-constrained health care systems. Consequently, accurately identifying adrenal lesions that do not require further investigation or follow-up is important.

In a large tertiary center study of adrenal incidentalomas, important predictors of malignancy were an unenhanced CT attenuation value of >20 Hounsfield units (HU) (odds ratio [OR], 28.40), androgen excess (OR, 27.67), detection during general cancer surveillance (OR, 11.34), size >4 cm (OR, 6.11), and male sex (OR, 3.06) (8). A threshold of >10 HU for unenhanced attenuation on CT has been reported in a meta-analysis of adrenal incidentalomas to have a sensitivity of 100% for the detection of malignancy (9). In contrast, imaging features that favor a benign adrenal lesion include homogeneity, with unenhanced attenuation <10 HU on CT, absent radioligand uptake or uptake less than the liver on [^18^F]fluorodeoxyglucose ([^18^F]FDG) positron emission tomography (PET) CT (PET-CT), loss of signal intensity (“signal drop out”) on out-of-phase sequences during chemical shift magnetic resonance imaging (MRI), and a relative washout of >58% on delayed contrast-enhanced CT (5). Furthermore, the presence of a high proportion of macroscopic fat (typically >50%) within an adrenal lesion is commonly used to support a radiological diagnosis of a benign myelolipoma (10).

European Society of Endocrinology adrenal incidentaloma guidelines recommend that in a patient with a homogenous, nonfunctioning adrenal lesion <4 cm in size, and unenhanced CT attenuation **≤**10 HU, no further investigation or follow-up is required. For homogeneous nonfunctioning adrenal lesions <4 cm size, but with attenuation values on unenhanced CT between 11 and 20 HU, either immediate further characterization with a second imaging modality (eg, chemical shift MRI) or interval imaging at 12 months is recommended. Early multidisciplinary team (MDT) review is recommended for adrenal lesions with any of the following characteristics: (1) unenhanced CT attenuation of 11 to 20 HU with a size >4 cm; (2) homogeneous lesions <4 cm with unenhanced CT attenuation >20 HU; or (3) heterogeneous lesions, regardless of size. In these scenarios, expert discussion helps guide decisions regarding further investigation and management. Adrenal lesions falling outside of these criteria should be considered for early surgical intervention. In contrast, adrenal lesions <1 cm in size, and without other concerning features, typically do not require investigation or follow-up (5).

A small number of case reports have recently described ACC developing several years after the diagnosis of an ipsilateral adrenal adenoma (11-14). In addition, cases of ACC arising within a preexisting adrenal adenoma have been reported (15-17). Here, we present 2 further cases identified through review of adrenal multidisciplinary team referrals at 2 tertiary centers in the United Kingdom, in which patients appeared to develop ACC several years after the initial detection of an adrenal lesion. Both patients met recommended criteria for discharge, based on low unenhanced CT attenuation, prolonged radiological stability over time, and reassuring features on an additional imaging modality. These cases raise the possibility—albeit rare—of progression from adrenal adenoma to carcinoma and underscore the need for improved biomarkers to inform long-term follow-up strategies in selected patients with incidentally discovered adrenal lesions.

## Methods

Cases were selected from a retrospective review of adrenal MDT records from 2021 through 2025 at Cambridge University Hospital NHS Foundation Trust (∼240 patients a year reviewed) and University Hospitals Birmingham NHS Foundation Trust (∼260 patients a year reviewed). Patients with a diagnosis of ACC were included if they had been: (1) reviewed at an adrenal MDT and (2) had previous imaging suggestive of an adrenal adenoma (lesion size <4 cm, unenhanced density ≤20 HU, lesion stability over time). Two cases were identified for detailed description, with ACC confirmed by either histopathology or by molecular imaging methods (Table 1). We excluded cases with prior incomplete radiological characterization and cases where the baseline unenhanced density was >20 HU.

**Table 1:** Two cases from two UK tertiary centers who had baseline radiological features in keeping with an adrenal adenoma (size < 4 cm and unenhanced density ≤20 HU) with prolonged dimensional stability on serial imaging, prior to a period of accelerated growth and diagnosis with ACC

Demographics				Identification and monitoring of adrenal adenoma				Apparent transformation to ACC				Treatment and Outcome
Case	Age at initial nodule detection	Sex	Other medical comorbidities	Initial imaging	Initial biochemical assessment	Time to ACC diagnosis	Interval imaging	Imaging at diagnosis of ACC	Hormone secretion	Histopathology	Metastases	
1	57	M	COPD, T2DM, OSA, Obesity	CT TAP with contrast: 30 mm homogeneous left adrenal nodule. Initial unenhanced HU not available.	Nonfunctioning	14 years	CT TAP at year 4: 37 mm, unenhanced HU −10 CT aortogram at year 5: 40 mm, unenhanced HU 4 MRI adrenal at years 12 and 14: stable size, signal drop out on chemical shift imaging	[^11^C]MTO PET-CT: 79 mm × 60 mm metomidate-avid left adrenal mass with metomidate-avid lung and nodal metastases	Mild autonomous cortisol secretion	N/A	Lung, mediastinal and hilar nodes	Best supportive care, died 2 months following diagnosis.
2	64	F	Asthma, HTN, Follicular lymphoma	CT colon with contrast: 23 mm × 28 mm right adrenal nodule. Initial unenhanced HU not available.	Not performed	11 years	Annual contrast-enhanced CT scans showing stable adrenal nodule [^18^F]FDG-PET at year 4: unenhanced HU 19.6, no tracer uptake [^18^F]FDG-PET at year 8: no tracer uptake	[^18^F]CETO PET-CT: 96 mm CETO-avid heterogeneous right adrenal lesion	Mild autonomous cortisol secretion	100 mm adrenocortical carcinoma; R0 resection; LWB—2 major and 2 minor criteria; Ki67 12%.	None	[^18^F]CETO PET-CT avid pulmonary metastases at 16 months postoperatively, commenced on mitotane.

Abbreviations: COPD, chronic obstructive pulmonary disease; CT TAP, computed tomography scan of thorax, abdomen, and pelvis; [^11^C]MTO-PET, [^11^C]metomidate positron emission tomography; [^18^F]CETO PET, para-chloro-2-[^18^F]fluoroethyletomidate positron emission tomography; [^18^F]FDG-PET, [^18^F]fluorodeoxyglucose positron emission tomography; HTN, hypertension; HU, Hounsfield units; LWB, Linn-Weiss-Bisceglia criteria; OSA, obstructive sleep apnea; T2DM, type 2 diabetes.

A literature review was performed on PubMed using the terms “adrenocortical cancer,” “ACC,” and “adenoma.” Case reports were included if they had serial adrenal imaging prior to diagnosis of ACC demonstrating prolonged dimensional stability. Case reports were categorized into those where initial radiological characterization demonstrated benign radiological features (lesion size <4 cm and unenhanced CT attenuation ≤20 HU and dimensional stability >1 year) (n = 4, Table 2) and those with either incompletely described or indeterminate nodules (lesion size <4 cm with unenhanced density >20 HU, but with dimensional stability >1 year) (n = 4, Table 3). These subcategories were included to investigate differences in the rate of growth between lesions that had baseline characteristics suggestive of a lipid-rich adenoma vs those with indeterminate radiological characteristics. Cases from case series were also included during the search.

**Table 2:** Four cases from the literature who had baseline radiological features in-keeping with an adrenal adenoma (size <4 cm and unenhanced density ≤20 HU) with prolonged dimensional stability on serial imaging, prior to a period of accelerated growth and diagnosis with ACC

Demographics				Identification and monitoring of adrenal adenoma				Apparent transformation to ACC				Treatment and outcome
Case	Age at initial nodule detection	Sex	Other medical comorbidities	Initial imaging	Initial biochemical assessment	Time to ACC diagnosis	Interval imaging	Imaging at diagnosis of ACC	Hormone secretion	Histopathology	Metastases at diagnosis	
Angelousi et al 2024 (11)	64	M	HTN	30-mm homogenous left adrenal lesion, HU <10	Nonfunctioning	13 years	Yearly CT for 3 years, then every 2 years. Lost to follow up for 4 years. CT scan at year 11 showing 30 mm lesion but 24 HU.	CT AP at year 13: 120 mm heterogeneous left adrenal lesion	Cortisol, androgens	Metastatic ACC; Ki67 70%, Weiss score not reported (from liver lesion biopsy)	Liver, lung	Treated with mitotane and etoposide, doxorubicin and cisplatin with partial response. Alive at 8 months after diagnosis.
Cristofolini et al 2024 (12)	73	M	Not reported	14-mm left adrenal lesion, HU 20	Nonfunctioning	5 years	6 monthly CT scans for 2 years.	CT AP at year 5: 145 mm left adrenal lesion	None	ACC, Ki67 5%; Weiss score 7	None	Left adrenalectomy and nephrectomy. No adjuvant therapy. No recurrence at 3 months postoperatively.
Parry et al 2024 (13)	70	F	T2DM, HTN, OA, AF, meningioma	8-mm right adrenal nodule, 3 HU	Nonfunctioning	7 years	Yearly CT for 5 years. CT at year 3 showing increase in density to 30 HU but stable size.	CT AP at year 7: 66 mm right adrenal lesion, HU 32	None	90-mm ACC; Ki67 20%; Weiss score 5	None	Open right adrenalectomy and partial hepatectomy. Local recurrence and metastasis, death 1 year after diagnosis.
Belmihoub et al 2017 (14)	71	M	Myelodysplasia, recurrent UTI	17-mm right adrenal nodule, HU 7.9	Nonfunctioning	13 years	CT at years 1, 3, and 5.	CT AP at year 15: 60-mm heterogeneous mass, HU 25	None	ACC; Ki67 30%; Weiss score 8	None	Right adrenalectomy. CT scan 5 months postoperatively showed local recurrence with liver, lung and peritoneal metastases. Died 2 months later.

Abbreviations: AF, atrial fibrillation; CT AP, computed tomography scan of abdomen and pelvis; EDP, etoposide; doxorubicin, and cisplatin; FDG-PET, fluorodeoxyglucose positron emission tomography; HTN, hypertension; HU, Hounsfield units (on unenhanced CT, unless otherwise specified); OA, osteoarthritis; T2DM, type 2 diabetes; UTI, urinary tract infection.

**Table 3:** Four cases from the literature who had indeterminate (<4 cm and unenhanced density >20 HU) or incompletely characterized adrenal lesions at baseline who demonstrated prolonged dimensional stability on serial imaging prior to accelerated growth and diagnosis with ACC

Demographics				Identification and monitoring of adrenal adenoma				Apparent transformation to ACC				Treatment and outcome
Case	Age at initial nodule detection	Sex	Other medical comorbidities	Initial imaging	Initial biochemical assessment	Time to ACC diagnosis	Interval imaging	Imaging at diagnosis of ACC	Hormone secretion	Histopathology	Metastases at diagnosis	
Gagnon et al 2020 (29)	32	F	Urolithiasis, IBS, fibromyalgia	29-mm left adrenal nodule, 31 HU	Nonfunctioning	10 years	MRI at year 2-24 mm, no loss of signal on out of phase imaging. CT at year 2: 25 mm, 20 HU. CT at year 4: 25 mm, 23 HU.	CT at year 10: 90-mm left adrenal lesion	Cortisol, androgens	120 mm ACC; Ki67 30%; Modified Weiss score 6	Lung, liver	Treated with mitotane and EDP. VUS found in *APC* gene.
Kohli et al 2021 (30)	61	F	HTN, Crohn disease, Osteopenia	20-mm left adrenal nodule, HU >10, absolute washout, 67% and relative washout >47%	Nonfunctioning	9 years	CT scan at year 1, 4, 5, and 7 stable	CT AP at year 9: 58 mm left adrenal lesion, 37 HU	Cortisol	80 mm ACC; Ki67 20%-30%; Weiss score not reported	None	Left adrenalectomy. Adjuvant radiotherapy and mitotane. Developed lung and peritoneal metastases. Died 15 months after diagnosis.
Aono et al 2022 (31)	68	F	HTN, RA	16-mm left adrenal nodule, 30 HU	Incomplete	9 years	CT scan at year 3 and year 5 stable. CT scan at year 8-30 mm, irregular.	CT at year 9: 54-mm heterogeneous left adrenal lesion, HU not reported	Cortisol	70-mm ACC; Ki67 20%; Weiss 4.	None	Adjuvant mitotane. Local recurrence at 18 months postoperatively treated with radiotherapy.
Ros et al 2024 (32)	74	F	HTN, IHD, PAD, AF, COPD, CKD3, osteoporosis, hypothyroidism	14-mm left adrenal nodule, 23 HU with absolute washout 84% and relative washout 71%. 6 mm left adrenal nodule 20 HU with absolute washout 67% and relative washout 43%.	Primary aldosteronism	7 years	CT scan year 5 both nodules stable size. FDG-PET mild uptake in both adrenal nodules. CT year 6: 25-mm left adrenal nodule	CT year 7: 35-mm left adrenal lesion, 40 HU	None	38-mm ACC; Ki67 50%-60%; Weiss 6	None reported	Adjuvant mitotane

Abbreviations: CKD, chronic kidney disease; COPD, chronic obstructive pulmonary disease; CT AP, computed tomography of the abdomen and pelvis; EDP, etoposide, doxorubicin, and cisplatin; HTN, hypertension; HU, Hounsfield units (on unenhanced CT, unless otherwise specified); IBS, irritable bowel syndrome; IHD, ischemic heart disease; PAD, peripheral arterial disease; RA, rheumatoid arthritis; VUS, variant of unknown significance.

### Case 1

**Figure 1:**
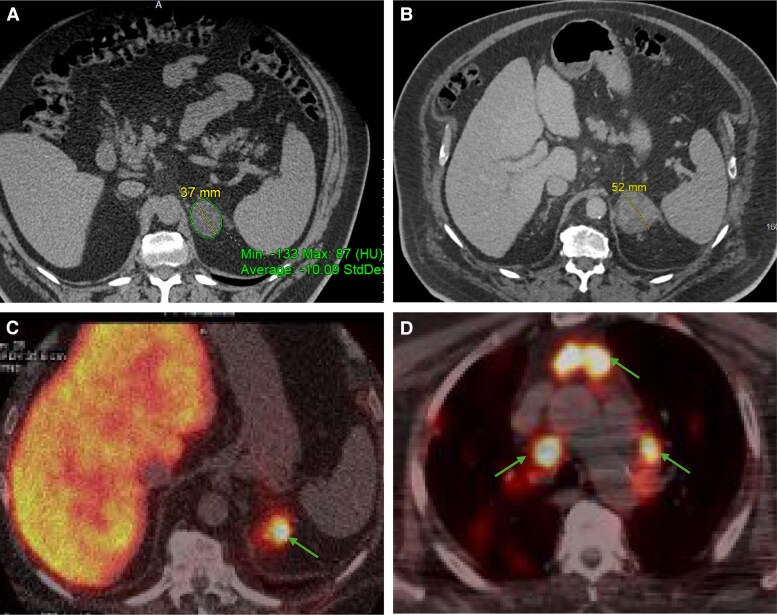
Imaging studies for case 1 showing characterization as a lipid-rich adenoma (A), followed by diagnosis of ACC (B-D). (A) Noncontrast CT performed at year 4 showing a 37-mm left adrenal nodule with density −10 HU. (B) Contrast-enhanced CT at year 14 showing an increase in lesion size to 52 mm. (C) [^11^C]Metomidate PET-CT showing an avidly enhancing left adrenal lesion (green arrow) with (D) multiple [^11^C]Metomidate avid thoracic nodal metastases (green arrows).

A man in his 60s was referred to the adrenal service for further assessment of a 40-mm left adrenal lesion seen on a CT pulmonary angiogram. Twelve years earlier, a 30-mm left adrenal lesion had been detected incidentally on a contrast-enhanced CT scan of the abdomen and pelvis, which was performed during an admission with meningitis. At the time, the lesion was considered most consistent with an adrenal adenoma, but no further clinical or radiological characterization was undertaken. Four years later (8 years prior to referral to the adrenal service), the lesion was formally characterized as a homogenous 37-mm nonfunctioning adenoma with unenhanced density of −10 HU and normal biochemical assessment (Fig. 1).

**Figure 2:**
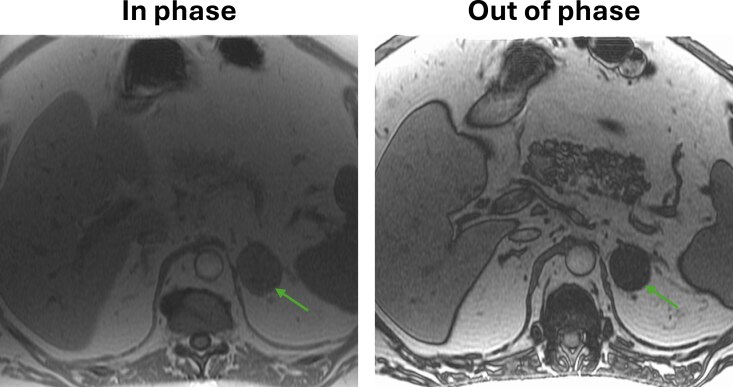
MRI of the adrenal glands performed for case 1 at 12 years after initial lesion detection, demonstrating signal drop out on out-of-phase chemical shift imaging in-keeping with a left-sided, lipid-rich adenoma (green arrows).

Following referral to the adrenal service, an MRI scan was performed which demonstrated a 40-mm left adrenal lesion with signal loss on chemical shift imaging (Fig. 2). There were no overt clinical features of hypercortisolism, but biochemical assessment was consistent with mild autonomous cortisol secretion (MACS) with a nonsuppressed overnight dexamethasone suppression test of 81 nmol/L (reference range (RR) < 50 nmol/L) and elevated late night salivary cortisol levels of 4.5 nmol/L and 4.6 nmol/L (RR < 4.3 nmol/L). In view of the lesion size and possible MACS, continued surveillance was advised and included a repeat MRI scan 2 years later that showed stable size (42 mm) and continued signal dropout in keeping with an adenoma. Given the patient's associated comorbidities, the adrenal MDT felt he would be a high-risk surgical candidate, and he was discharged with advice for cardiovascular risk optimization. At this stage, his left adrenal lesion had been stable for 14 years (Table 1).

However, 8 months later, a CT pulmonary angiogram performed for dyspnea showed multiple scattered lung nodules and enlarged thoracic nodes concerning for metastatic malignancy. In addition, the left adrenal lesion had increased in size from 42 × 35 mm to 52 × 49 mm (Panel B, Fig. 1). [^18^F]FDG PET-CT showed the left adrenal lesion was now heterogeneous, and both the adrenal and pulmonary lesions demonstrated high [^18^F]FDG uptake. [^11^C]metomidate PET-CT (using the radioligand [^11^C]metomidate ([^11^C]MTO), which shows highly selective binding to the adrenocortical enzymes CYP11B1 and CYP11B2) was performed 3 months later and revealed a 79-mm [^11^C]MTO-avid left adrenal lesion, with high tracer uptake also seen in multiple pulmonary and thoracic nodal metastases, consistent with metastatic ACC (Fig. 1). Following multidisciplinary team discussion, a decision was made to pursue best supportive care in view of the patient's poor performance status. He died 2 months later.

### Case 2

A woman in her 70s with a background of follicular lymphoma was referred to the adrenal service after a 37-mm right adrenal lesion was identified on contrast-enhanced CT colonography performed for altered bowel habit. The lesion had first been noted 11 years earlier on a contrast-enhanced CT scan of the abdomen. Over the subsequent 11 years, annual contrast-enhanced CT scans performed as part of her lymphoma surveillance program demonstrated slow interval growth of the adrenal lesion, with an overall increase in size of 9 mm (ie, <1 mm per year). In addition, 1 [¹⁸F]FDG PET-CT scans performed at years 4 and 8 had shown very low [¹⁸F]FDG uptake within the right adrenal lesion, which had also been found to have an attenuation value of 19.6 HU on unenhanced CT at the time of the first [¹⁸F]FDG scan. Taken together, these findings were considered most consistent with an adrenal adenoma.

**Figure 3:**
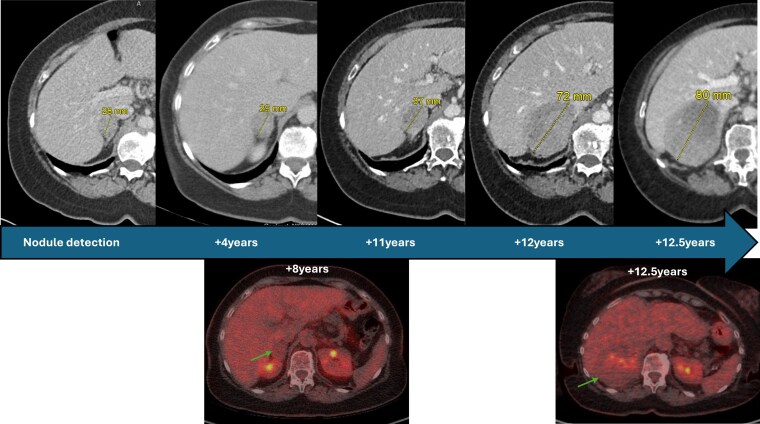
A timeline of portal phase intravenous contrast CT scans performed for case 2 demonstrating size stability of the right adrenal lesion for 11 years, followed by an accelerated growth trajectory. The bottom row shows 2 fused axial FDG-PET CT images demonstrating the non-FDG-avid homogenous right adrenal nodule at 8 years after detection, followed by a large right adrenal lesion with a heterogeneous pattern of FDG avidity at 12.5 years after detection (green arrows).

On review in the adrenal service, MACS was suspected with the finding of a serum cortisol of 109 nmol/L (RR < 50 nmol/L) following a 1-mg overnight dexamethasone suppression test (with adequate serum dexamethasone level of 6.1 nmol/L). Two late night salivary cortisol tests and 24-hour urinary free cortisol measurements were unremarkable. However, at the time of referral, the 37-mm right adrenal lesion now exhibited an attenuation value of 33 HU on unenhanced CT. In view of the <1-mm/year growth over an 11-year period and very low level [¹⁸F]FDG uptake on 2 occasions, the adrenal MDT felt the lesion was still most likely to represent a lipid-poor adenoma. A urine steroid profile was requested that identified relative increases in the 17-hydroxyprogesterone metabolite pregnanetriol and the progesterone metabolite pregnanediol, alongside a relative increase in one of the ACC markers 5-pregnene-3a,16a,20-triol. However, this report was not considered conclusive for the presence of ACC and, taking into account the size of the adrenal lesion (<4 cm) and stability over time, after discussion with the patient, a decision was taken to continue with regular surveillance imaging. Interval CT 12 months later revealed a significant increase in size of the adrenal lesion to 57 × 72 mm (Fig. 3). Given the patient's history of an extra-adrenal malignancy, and the importance of distinguishing between an adrenocortical and a nonadrenocortical lesion, para-chloro-2[^18^F]fluoroethyletomidate ([^18^F]CETO) PET-CT (which, similar to [^11^C]MTO PET, specifically localizes tissues expressing CYP11B1 and CYP11B2) was performed and showed heterogeneous, avid [^18^F]CETO uptake in the right adrenal lesion confirming its adrenocortical origin. Repeat [^18^F]FDG PET-CT now demonstrated heterogeneous high uptake in the right adrenal lesion. There were no other [^18^F]CETO or [^18^F]FDG-avid lesions suggestive of metastatic disease. She was referred for right adrenalectomy and histology confirmed a 100-mm adrenocortical carcinoma (Table 1).

### Cases from the literature

Four case reports were identified describing patients with ACC who had evidence of a preexisting ipsilateral adrenal lesion that was deemed at baseline to be consistent with an adenoma (lesion size <4 cm with unenhanced CT attenuation ≤20 HU) (Table 2). Of note, 3 of the 4 cases documented unenhanced CT attenuation values of <10 HU on initial imaging and all cases reported stability in lesion size on serial CT surveillance over a 5- to 13-year interval. None of the cases had evidence of hormonal hypersecretion at baseline assessment, but 1 subsequently developed cortisol excess and a further case developed mixed cortisol and androgen excess. Importantly, the cases reported by Angelousi et al (11). and Parry et al (13). both showed an increase in unenhanced attenuation during CT surveillance (<10 to 24 HU and 3 to 30 HU, respectively) prior to demonstrating an increase in lesion size. This mirrors the observed change in unenhanced CT attenuation from 19.6 to 33 HU prior to significant dimensional change in Case 2 reported here.

Four further cases reporting indeterminate (lesion size <4 cm and unenhanced attenuation >20 HU) or incompletely characterized adrenal lesions, which exhibited a prolonged period of absent or indolent growth prior to diagnosis of ACC are summarized in Table 3. All 4 cases had prolonged size stability on serial imaging and 2 of the 4 cases also underwent immediate further characterization with reassuring absolute (>60%) and relative (>40%) washout on delayed contrast-enhanced CT.

Two studies have focused on the identification of preexisting adrenal lesions in patients subsequently found to have an ACC. In a retrospective single-center study by Nogueira et al., 20 of 422 patients (4.7%) with ACC had radiological evidence of adrenal nodules 5 months or more prior to diagnosis. Six of 20 patients were reported as having a latent period of relative stability (defined by the authors as minimal growth), with 1 patient demonstrating dimensional lesion stability for 8 years prior to an accelerated growth phase. However, information regarding the serial interval imaging performed was not reported for individual patients and the reported rate of growth calculated by the authors was presented as an average (representing the increase in size between initial imaging and imaging at diagnosis of ACC/time in years), which does not reflect the variable growth rate trajectories of individual patients (18). In a second retrospective study looking at delayed diagnosis of ACC, Ozsari et al. identified 25 of 439 patients with preexisting cross-sectional images for review and found 20 of 25 patients had preexisting adrenal masses on imaging >3 months prior to ACC diagnosis. Again, this study demonstrated the variable growth trajectories of these preexisting lesions. One patient who had an initial lesion size of 25 mm and unenhanced density of 17 HU showed radiological stability at 24 and 48 months, followed by accelerated growth and eventual diagnosis of a 53 mm ACC at 72 months. Other reported patients had a significantly more rapid growth trajectory (19). However, in both case series, no patients had a baseline unenhanced density of <10 HU (18, 19). Because of the incomplete phenotypical data, these cases reported in Nogueira et al. and Ozsari et al. have not been included in Table 3.

Three examples of ACC detected within a hyperplastic or adenomatous lesion on histopathological examination have also been reported, with 2 cases undergoing comparative genetic analysis of the different components (15-17). Bernard et al. reported an incidentally detected 55-mm adrenal lesion associated with MACS, which upon pathological examination was found to contain a 22-mm central malignant component (Weiss score 5, Ki67 > 10%) surrounded by a rim of tissue with a benign appearance (Weiss score 0, Ki67 < 1%). The central malignant component was associated with p53 loss of heterozygosity and overexpression of IGF2 in contrast to the benign-appearing peripheral tissue (16). Dybal et al. reported a 45-mm right adrenal lesion associated with MACS, which on histopathological examination was found to comprise a central 27-mm core of ACC (Weiss score 5, Ki67 40%) embedded within a benign clear-cell rich nodule. Although the benign-appearing region of the nodule had heterozygous deletion of *CDKN2A* and an *ARID1A* exon 20 variant, the adjacent ACC cells had somatic homozygous deletions of *CDKN2A*, *CDKN2B* and *MTAP*, *TERT* amplification, *CTNNB1* mutation, *ARID1A* exon 20 variation, and *ARID1A* loss of heterozygosity. The patient had previously undergone a contralateral adrenalectomy for an adenoma associated with cortisol excess, but none of the bilateral macronodular adrenal hyperplasia (BMAD) associated germline mutations had been identified (15)

## Discussion

We report 2 patients from 2 tertiary centers within the United Kingdom (Table 1) and a further 4 cases from the literature (Table 2) who demonstrated benign radiological characteristics at baseline, but were subsequently diagnosed with ACC. All 6 patients had serial adrenal imaging demonstrating dimensional stability for up to 14 years. Three of the 6 patients demonstrated an increase in unenhanced density prior to an increase in growth rate and 1 patient had an abnormal urine steroid profile. A further 3 examples of ACC embedded within a hyperplastic or adenomatous lesion were also identified in the literature.

We also identified 4 case reports of indeterminate adrenal nodules that showed prolonged lesion stability or indolent growth (the fastest being ∼3 mm/year) for up to 10 years prior to an accelerated growth phase and diagnosis with ACC (Table 3). Several additional examples of ACCs with a prolonged period (up to 8 years) of dimensional stability were identified within 2 case series but were missing individualized case information regarding radiological surveillance.

The mean time to ACC diagnosis for patients with benign radiological characteristics at baseline (lesion size <4 cm and unenhanced density ≤20 HU; Tables 1 and 2) was 10.5 years (range, 5-14 years), and for patients with indeterminate radiological characteristics at baseline (lesion size <4 cm and unenhanced density >20 HU or incompletely characterized; Table 3) that was 8.75 years (range, 7-10 years). The difference between these 2 groups in the average time to ACC diagnosis was not statistically significant on Student *t*-test analysis (*P* = .39). The growth trajectory of an adrenal lesion prior to diagnosis with ACC is evidently variable, with some lesions demonstrating dimensional stability over several years.

The adenoma to carcinoma sequence is well established for colorectal cancer. Mutation in the *APC* tumor suppressor gene, which is a component of the β-catenin (CTNNB1) destruction complex, is widely accepted as a common initiating event for colorectal adenoma development. Different pathways have been established for the colorectal adenoma-carcinoma transition following this, including that of chromosomal instability, driven primarily by Wnt signaling pathway activation, KRAS activation and TP53 mutations, or the microsatellite instability pathway where loss of mismatch repair gene function causes progressive accumulation of somatic base pair mutations (20). This sequential transition has not been proven in the adrenal gland, though the 6 cases that we describe and 3 additional reports of adrenocortical carcinoma embedded within an adenoma indicate that it may be possible. Most case series of adrenal incidentalomas, however, do not report progression to malignancy (2), and it is likely that it remains a rare event.

Transcriptome analysis of adrenocortical tumors shows discrete clustering of adrenocortical tumors into 3 subtypes, namely C1a, C1b, and C2, which correspond to an aggressive group of ACC with high expression of cell-cycle genes, a less aggressive ACC group with an immune signature, and a high adrenal differentiation group representing adenomas, respectively (21-24). Mutations in β-catenin (*CTNNB1*) are present in up to 30% of both adrenocortical adenomas and carcinomas, suggesting that the constitutive activation of the Wnt/β-catenin signalling pathway may be an early step in tumorigenesis (25, 26). *Notch* mutations are also commonly reported in both adrenocortical adenoma and carcinoma (27). As seen in the reported cases of ACC embedded within a benign lesion, IGF2 overexpression, however, is specific to ACC, and ACCs have a higher number of genomic alterations (15, 16, 27). Mouse models suggest that both altered β-catenin and IGF-2 expression may be required to transition from adrenal hyperplasia to tumorigenesis (28). In ACC, key driver genes are *CTNNB1*, *TP53*, *CDKN2A*, *RB1,* and *MEN1,* with a study also identifying *ZNRF3* as a tumor suppressor gene altered in 21% of cases (22). Interestingly, Jouinot et al. studied a subgroup of histopathologically heterogeneous ACCs and demonstrated that the transcriptome analysis of the benign-appearing components was concordant with that of the more histopathologically aggressive parts (23). Further research is required to understand if, and how, the stepwise progression from adrenal adenoma to carcinoma occurs, or if these cases represent independent or collision tumour events.

The potential rare occurrence of transformation from adrenal adenoma to ACC poses a challenge for clinicians, who need to balance increased surveillance for early detection of ACC with the risks of iatrogenic harm from radiation exposure or incidental findings, and the financial burden of repeated abdominal imaging in resource-limited settings. It is important to emphasize that the 2 cases reported from our tertiary centers represent approximately 0.1% of all referrals to 2 large tertiary center adrenal MDTs over a 4-year period.

However, our cases demonstrate that lesion size stability can be falsely reassuring. One observation from this case series and literature review was that for 3 of our 6 reported patients with baseline unenhanced density of <10 HU, an increase in unenhanced density was seen prior to an increase in size. While this change in unenhanced density may lead the MDT to consider further interval imaging rather than discharge, an important caveat is that unenhanced density can only be applied to radiologically homogenous adrenal lesions and can be a subjective measurement, relying on an experienced radiologist. Case 2 also demonstrates the utility of urine steroid profile assessment in the evaluation of indeterminate adrenal nodules, though further prospective studies to evaluate the utility of urine steroid profile for indeterminate nodules of <4 cm size are needed.

Many patients with adrenal lesions that are characterized as radiologically indeterminate (unenhanced HU >20, heterogeneous appearance) will be recommended for an adrenalectomy. However, patients that are managed with radiological surveillance due to comorbidities or personal preference should be counseled that ACC remains a possibility even if the lesion is stable for >12 months. In this case series and literature review, we have reported 4 cases that were stable on serial imaging for >12 months (Table 3) and for whom the mean time to accelerated growth was 8.75 years. This suggests that if surveillance of radiologically indeterminate lesions is chosen as the preferred option patients should be counseled that exclusion of an ACC may require many years of radiological surveillance.

A limitation of this retrospective literature review is that we are likely to have selected the most indolent cases of ACC due to our predefined criteria of lesional stability for >12 months. It is not possible to accurately quantify the proportion of patients with radiologically indeterminate lesions who go on to develop ACC after a prolonged period of stability of >12 months, as most patients would either be offered adrenalectomy after baseline assessment or would be discharged after 12 months of follow up. A further limitation of this case series is that a histopathological diagnosis was not available for case 1; however, the authors believe that the presence of adrenocortical tissue in the lungs as demonstrated by [^11^C]MTO uptake on PET-CT is most in keeping with a diagnosis of metastatic ACC.

In conclusion, adrenal lesions with baseline benign radiological characteristics can very rarely transpire to be ACC. Prolonged lesional stability cannot be considered a reassuring feature in isolation when assessing radiologically indeterminate adrenal lesions in the adrenal MDT and this case series and literature review demonstrates that a small risk of ACC remains even after >10 years of dimensional stability. Further research to understand the molecular mechanisms underpinning a possible adrenal adenoma to carcinoma transformation would be valuable and this article highlights the need for additional prognostic biomarkers for stratifying risk in radiologically indeterminate lesions.

## Data Availability

Data sharing is not applicable to this article as no datasets were generated or analyzed during the current study.

## Abbreviations

ACC: adrenocortical carcinoma
CT: computed tomography
HU: Hounsfield unit
MACS: mild autonomous cortisol secretion
MDT: multidisciplinary team
MRI: magnetic resonance imaging
OR: odds ratio
PET: positron emission tomography
RR: reference range
